# Cardiovascular Biomarkers in Cardio-Oncology: Antineoplastic Drug Cardiotoxicity and Beyond

**DOI:** 10.3390/biom14020199

**Published:** 2024-02-07

**Authors:** Umberto Attanasio, Elena Di Sarro, Lucia Tricarico, Daniela Di Lisi, Giuseppe Armentaro, Sofia Miceli, Francesco Fioretti, Martino Deidda, Michele Correale, Giuseppina Novo, Angela Sciacqua, Savina Nodari, Christian Cadeddu, Carlo Gabriele Tocchetti, Alberto Palazzuoli, Valentina Mercurio

**Affiliations:** 1 Department of Translational Medical Sciences, Federico II University, Via Sergio Pansini 5, 80131 Naples, Italyelena.disarro@gmail.com (E.D.S.); cgtocchetti@gmail.com (C.G.T.); 2 Cardiology Unit, Department of Medical and Surgical Sciences, University of Foggia, Viale Pinto 1, 71122 Foggia, Italy; lucia.tricarico.lt@gmail.com (L.T.); michele.correale@libero.it (M.C.); 3 Department of Health Promotion, Mother and Child Care, Internal Medicine and Medical Specialties (PROMISE), University of Palermo, Piazza delle Cliniche 2, 90127 Palermo, Italy; danydilis@hotmail.it (D.D.L.); giuseppina.novo@policlinico.pa.it (G.N.); 4 Division of Cardiology, University Hospital Paolo Giaccone, Via del Vespro 129, 90127 Palermo, Italy; 5 Department of Medical and Surgical Sciences, University Magna Græcia of Catanzaro, Campus Universitario di Germaneto, V.le Europa, 88100 Catanzaro, Italy; giuseppearmentaro91@gmail.com (G.A.); sofy.miceli@libero.it (S.M.); sciacqua@unicz.it (A.S.); 6 Cardiology Section, Department of Medical and Surgical Specialties, Radiological Sciences and Public Health, Spedali Civili Hospital and University of Brescia, Piazzale Spedali Civili 1, 25123 Brescia, Italy; franc.fioretti@gmail.com (F.F.); savina.nodari@unibs.it (S.N.); 7 Department of Medical Sciences and Public Health, University of Cagliari, 09042 Monserrato, Italycadedduc@unica.it (C.C.); 8 Interdepartmental Center of Clinical and Translational Sciences (CIRCET), Federico II University, Via Sergio Pansini 5, 80131 Naples, Italy; 9 Interdepartmental Hypertension Research Center (CIRIAPA), Federico II University, Via Sergio Pansini 5, 80131 Naples, Italy; 10 Center for Basic and Clinical Immunology Research (CISI), Federico II University, Via Sergio Pansini 5, 80131 Naples, Italy; 11 Cardiovascular Diseases Unit, Cardio-thoracic and Vascular Department Le Scotte Hospital, University of Siena, Strada delle Scotte 14, 53100 Siena, Italy; palazzuoli2@unisi.it

**Keywords:** troponin, natriuretic peptides, biomarkers, omics science, cancer, cardiotoxicity, heart failure, cardiac dysfunction, multiple myeloma

## Abstract

Serum biomarkers represent a reproducible, sensitive, minimally invasive and inexpensive method to explore possible adverse cardiovascular effects of antineoplastic treatments. They are useful tools in risk stratification, the early detection of cardiotoxicity and the follow-up and prognostic assessment of cancer patients. In this literature review, we aim at describing the current state of knowledge on the meaning and the usefulness of cardiovascular biomarkers in patients with cancer; analyzing the intricate relationship between cancer and cardiovascular disease (especially HF) and how this affects cardiovascular and tumor biomarkers; exploring the role of cardiovascular biomarkers in the risk stratification and in the identification of chemotherapy-induced cardiotoxicity; and providing a summary of the novel potential biomarkers in this clinical setting.

## 1. Introduction

Cardio-oncology is a discipline that studies the relationship between cancer and cardiovascular diseases (CVDs), and it is mostly focused on the prevention and management of cardiovascular damage resulting from anticancer therapies [1]. The possible cardiotoxic events induced by anticancer treatments include myocardial dysfunction, heart failure (HF), coronary artery disease, valvular disease, arrhythmias, pericardial disease, hypertension and thrombolytic events. In addition to the cardiotoxic effects of oncological therapies, it is believed that cancer and HF are linked by a bidirectional relationship, where one disease favors the other [2,3]. Serum biomarkers represent a reproducible, sensitive, minimally invasive and inexpensive method to explore these effects. They are useful tools in risk stratification, the early detection of cardiotoxicity, follow-up and prognostic assessment [2,4]. They may be good tools to identify patients at high risk of adverse cardiovascular effects before the initiation of therapy and to detect subclinical diseases during active therapy in combination with imaging, identifying patients who should receive cardioprotective therapies [5,6]. Troponins and natriuretic peptides have garnered the broadest evidence base for cardiotoxicity risk prediction, but other markers are being investigated. However, further studies are needed to assess the diagnostic and prognostic roles of other potential biomarkers, including inflammatory and other novel markers. This review article aims at describing the current state of knowledge on the meaning and the usefulness of cardiovascular biomarkers in patients with cancer; analyzing the intricate relationship between cancer and cardiovascular disease (especially HF) and how this affects cardiovascular and tumor biomarkers; exploring the role of cardiovascular biomarkers in the risk stratification and in the identification of chemotherapy-induced cardiotoxicity; and providing a summary of the novel potential biomarkers in this clinical setting.

## 2. Cardiovascular Biomarkers: Troponins and Natriuretic Peptides

A biomarker, a biological molecule detected in blood, body fluids or tissues, is linked to vital parameters and imaging tests. These markers play a crucial role in revealing disease characteristics, serving as indicators of the risk and disease state and predicting outcomes, including the rate of progression [7]. Cardiac biomarkers, such as troponin and natriuretic peptides (NPs), play pivotal roles in assessing heart health, offering valuable insights into myocardial damage and HF.

NPs, notably brain natriuretic peptide (BNP) and atrial natriuretic peptide (ANP), serve as widely utilized biomarkers in HF. Synthesized as prohormones, they undergo cleavage into active hormones (BNP and ANP) and inactive forms (NT-proBNP and MR-proANP). While BNP and ANP have short circulating half-lives, NT-proBNP and MR-proANP persist for longer, with renal clearance [8,9]. Considered the gold-standard biomarkers in HF, BNP and NT-proBNP receive Class IA recommendation in the guidelines of major societies, including the American Heart Association (AHA) and European Society of Cardiology (ESC) [10,11]. However, clinical interpretation is influenced by factors such as obesity, which lowers serum levels, and various conditions like age, heart diseases, valve disorders, atrial fibrillation and renal failure, which increase plasmatic concentrations [12,13]. BNP and NT-proBNP have similar clinical value for the evaluation of cardiac function, although NT-proBNP is more stable and does not appear to be affected by changes in anticoagulants, collection containers, body position or circadian rhythm [14]. They offer a non-invasive means to estimate intracardiac filling pressures and end-diastolic wall stress, enhancing the diagnostic accuracy when combined with clinical assessment, ECG, chest X-ray and echocardiography [11,15]. Several randomized clinical trials support the additional use of BNP or NT-proBNP concentrations, leading to improved medical and economic outcomes [16,17]. BNP and N-terminal pro-B-type natriuretic peptides (NT-proBNPs) are biomarkers of long-term cardiovascular dysfunction in asymptomatic patients [18,19,20,21] and represent an important screening tool for patients presenting with dyspnea during anticancer treatment. High diagnostic accuracy is observed in discriminating HF from other causes of dyspnea, with NP concentrations correlating with the likelihood of HF. The optimal cut-off concentrations for acute and chronic HF differ, with higher thresholds in cases of acute dyspnea [11]. NPs exhibit high prognostic accuracy in various conditions, including HF hospitalization, myocardial infarction, valvular heart disease, atrial fibrillation and pulmonary embolism. They also track variations in myocardial stress and dysfunction, making them valuable in conditions like Takotsubo syndrome and during cancer treatment. Myocardial ischemia triggers NP expression independently of mechanical stress, highlighting their role in HF worsening [11,22].

NPs are valuable markers for cardiotoxicity assessment. They can identify acute cardiotoxicity, particularly within 24 h of exposure to anthracycline chemotherapy [23]. While NPs are useful for HF screening in cancer patients with dyspnea, a cut-off value of 100 ng/L for NT-proBNP has high sensitivity [24,25]. Several studies indicate that elevated baseline NT-proBNP in cancer patients is a significant predictor of mortality risk [26,27,28,29].

Troponins are biomarkers that have always been used to diagnose acute coronary syndromes, but have proven to be useful in identifying cardiotoxicity. There are three types of troponins: troponin I, troponin T and troponin C. Cardiac troponin T (cTnT) and cardiac troponin I (cTnI), released during cardiac muscle cell injury, are heart-specific markers, but not disease-specific. Increased levels can be found in various conditions, both physiological (i.e., physical or psycho-emotional stress) and pathological, including chronic HF, diabetes, arterial hypertension, inflammatory heart disease, pulmonary embolism, chronic renal failure and sepsis. The methods of determining cTnT and cTnI have been continuously improved, increasing their analytical sensitivity and specificity [30]. Highly sensitive (hs) immunoassays are now available to determine hs-cTnT and hs-cTnI concentrations, detecting very low but diagnostically significant concentrations of circulating cardiac troponins, especially to identify subclinical cardiac damage [31,32,33]. Small changes in plasma high-sensitivity cTnI (hs-cTnI) below the 99th centile have prognostic value in various heart-related conditions. Temporal changes in high-sensitivity cardiac troponin concentrations help to differentiate acute from chronic cardiomyocyte injury, with a near-linear association between cTnT/cTnI concentrations and the risk of developing clinical HF, hospitalization, atrial fibrillation and death. Managing cardiomyocyte injury requires individualization based on dominant mechanisms, though this can be challenging [34].

In 2020, a meta-analysis of 61 trials involving 5691 adult cancer patients revealed that anticancer therapy often leads to an increase in troponin levels (OR 14.3, 95% CI 6.0–34.1; *n* = 3049). Elevated troponins were associated with a higher risk of left ventricular dysfunction (LVD) (OR 11.9, 95% CI 4.4–32.1; *n*  =  2163) [35]. This underscores the potential of troponin assessment in identifying patients at risk for cardiotoxicity during cancer treatment. Moreover, beta-blockers, but not candesartan or low-dose enalapril, were found effective in preventing troponin rises when used as a primary prevention strategy in two independent prospective trials, emphasizing the role of troponin monitoring in evaluating the response to cardioprotective treatment [36,37,38]. The combination of these biomarkers provides a comprehensive evaluation of cardiac function, aiding clinicians in making informed decisions about diagnosis, risk stratification and treatment planning. Their distinct specificities contribute to a more nuanced understanding of cardiac conditions, enhancing the overall effectiveness of cardiovascular care.

## 3. Cardiovascular Biomarkers in Cancer Patients vs. Tumor Biomarkers in Heart Failure Patients

The recent investigations on the possible interplay between cardiovascular biomarkers and cancer have unveiled a complex relationship, challenging the traditional perspectives on the exclusive association of cardiac biomarkers, such as troponin and natriuretic peptides, with some cardiovascular diseases or with the development of cancer therapy-related cardiovascular toxicity (CTRCD). In fact, even though the most recent recommendations on the management of patients at risk of developing CTRCD suggest the broad use of troponin and natriuretic peptides to stratify cardiovascular risk (at baseline) and to identify the early development of toxicity (during follow-up) [39], some evidence suggests that the circulating levels of NPs and troponins can be elevated in patients with neoplasms, even prior to the introduction of cancer therapy, with no evidence of an abnormal cardiac status [26]. Indeed, it is known that malignant cells are able to produce vasoactive peptides, such as vasopressin [40] or endothelin-1 [41], as well as cardiac neurohormones, like atrial natriuretic peptide [42] and B-type natriuretic peptide (BNP) [43,44]. Importantly, BNP elevation cannot be always explained by the presence of an underlying cardiac or hemodynamic condition that would typically affect BNP levels [45]. Moreover, the levels of troponin T (TnT) and troponin I (TnI) have been recently demonstrated to be elevated in cancer patients [46,47], being the former also associated with a worse prognosis [46]. The evidence that such cardiovascular biomarkers tend to increase proportionally with the advancing tumor stage, even without other signs of cardiac damage or dysfunction [26], may reinforce the idea that, in some cases, the elevation of these biomarkers could be hypothesized to be driven by the neoplastic disease, rather than the presence of an underlying cardiopathy or the occurrence of CTRCD. Certainly, this should not discourage the clinical use of these biomarkers according to the most recent guidelines [39], especially taking into account that they seem to be useful in the prediction of prognosis [26,46]. Of note, even though elevated levels of cardiovascular biomarkers were found in subjects without evidence of manifest cardiac involvement, the association between late-stage cancer, cancer cachexia and cardiac wasting has been extensively described and may represent a possible explanation for this phenomenon [48,49,50].

Conversely, a reciprocal relationship may be identified within cardiovascular conditions, specifically HF, presenting elevated levels of tumor biomarkers [51]. In fact, it has emerged that patients affected by HF present with high serum levels in several biomarkers presumed to be tumor-related, such as CA19-9, CA125 and human epididymis protein 4 (HE4) [51,52,53,54,55,56]. The possible interconnections between HF and cancer extend far beyond HF being a manifestation of CTRCD, and they have been deeply investigated in the last few decades [57,58,59,60,61,62,63]. Interestingly, an analysis of 2079 patients affected by HF in the BIOSTAT-CHF cohort [64] proved that the blood levels of five out of the six tumor biomarkers that they investigated (CA125, CA15-3, CA19-9, CEA and CYFRA 21-1) were significantly correlated with all-cause mortality, while CA125 also showed a strong correlation with HF hospitalization risk, and CYFRA 21-1 had equivalent predictive utility for all-cause mortality when compared with NTproBNP levels [51]. In particular, CA125 is probably the most investigated tumor biomarker in this setting, having shown prognostic value in different cardiac settings, being elevated in response to both congestion and inflammatory stress conditions [65,66,67,68,69]. This tumor biomarker is also associated with hospitalization, a worse prognosis and elevated NTproBNP serum levels [65,66,67,68,69]. Likewise, in recent studies, HE4, which is a serum biomarker currently used to monitor the recurrence of epithelial ovarian cancer, has been demonstrated to be strongly associated with HF and it seems to represent an independent predictor of HF outcomes [70,71,72].

Thus, the associations between established tumor biomarkers and indices of HF severity, along with their independent prognostic value for the HF outcomes of these biomarkers, and between classic cardiovascular biomarkers and cancer patient prognosis, may suggest the presence of dysregulated pathophysiologic pathways common to both cancer and cardiovascular diseases [61]. As already mentioned, the existent correlation between these two conditions is well recognized in the scientific literature and, remarkably, it has been highlighted that cancer and cardiovascular disease share many risk factors, as well as a common underlying inflammatory condition [57,58,59,61,62,73]. Of note, this chronic phlogistic state has been also observed in clinical practice and reported in the literature, with the help of serum inflammatory markers such as C-reactive protein (CRP) and proinflammatory cytokines such as interleukin 6 (IL-6) in both cancer and HF [19,74,75,76,77,78]. As highlighted for the other biomarkers, IL-6 seems to play a role in predicting prognosis in HF patients [79], and it was also found, together with CRP, to be proportionally elevated to the cardiovascular peptides (such as NTproBNP) in a population of 555 individuals with different types and stages of cancer that had not yet undergone chemotherapy; in addition, similarly to the trend of cardiac biomarkers, IL-6 serum levels were found to be higher in patients with more advanced stages of cancer [26].

To summarize, the intricate interplay between cardiovascular biomarkers and cancer extends beyond their traditional roles, revealing a complex relationship influenced by systemic inflammation and shared risk factors and pathophysiological pathways. These biomarkers, while often utilized to detect potential cardiac toxicity from cancer therapies, can also rise independently, even before chemotherapy, being sometimes able to stratify risk and predict prognosis. Such a bidirectional association, where cancer patients exhibit elevated cardiovascular biomarkers and cardiac patients present increased levels of tumor biomarkers, underscores the need for a deeper understanding of these markers in the context of the more precise and targeted use of these tools in both clinical scenarios.

## 4. Role of Cardiovascular Biomarkers in the Risk Stratification and in the Identification of Chemotherapy-Induced Cardiotoxicity in Cancer Patients

In recent years, important advances have been made in the field of cardio-oncology [39]. The ESC Guidelines on Cardio-Oncology recommend cardiovascular (CV) toxicity risk stratification before starting potentially cardiotoxic cancer therapy [39,80]. Baseline CV risk stratification in cancer patients is important in order to prescribe cardioprotective treatment before starting antineoplastic treatment when needed, to schedule a cardiology referral before treatment and to plan the most appropriate surveillance program during and after treatment [39]. Cardiotoxicity risk is a dynamic variable related not only to traditional cardiovascular risk factors but also to treatment-related factors; therefore, it is advisable to conduct its assessment by using a dedicated cancer patient tool such as the HFA-ICOS risk score [39,80]. Few studies have so far validated this score in patients with solid or hematological tumors [81,82]. The HFA-ICOS risk score takes into consideration lifestyle risk factors, demographic and CV risk factors, previous CV diseases, previous and concomitant cardiotoxic cancer treatment and baseline cardiac biomarkers (elevated baseline troponin—Tn, elevated baseline brain natriuretic peptide—BNP or NT-proBNP) in patients receiving six of the most frequently used anticancer treatments [80]. Monitoring biomarkers, and particularly Tn and BNP/NT-proBNP, during cancer treatment aids in the early diagnosis of cardiotoxicity in cancer patients [34].

Symptomatic CTRCD is defined by the presence of HF symptoms; the diagnosis of asymptomatic CTRCD is based on the left ventricular ejection fraction (LVEF) reduction and/or relative decline in global longitudinal strain (GLS) and/or a new rise in cardiac biomarkers (cTnI/cTnT gammaGT; 99th percentile, BNP ≥ 35 pg/mL, NT-proBNP ≥ 125 pg/mL, or a new significant rise from baseline beyond the biological and analytical variations of the assay used) [39]. Several studies have shown the prognostic role of monitoring cardiac troponin. In particular, Cardinale et al. demonstrated, in patients treated with high-dose anthracyclines, that persistently negative troponin is able to identify low-risk patients who do not need close echocardiographic monitoring, while a persistent troponin elevation preceded an LVEF drop and identified high-risk patients [83,84,85]. In patients treated with trastuzumab, a troponin increase identified patients at risk of non-reversible left ventricular dysfunction [86]. In a recent meta-analysis, Michel et al. confirmed the predictive role of a troponin increase after anthracycline-based chemotherapy or human epidermal receptor 2 (HER2) inhibitor therapy for the development of left ventricular dysfunction.

Petricciulo et al. showed that, in patients treated with immune checkpoint inhibitors (ICI), baseline hs-TnT predicted a composite cardiovascular endpoint (cardiovascular death, stroke or transient ischemic attack, pulmonary embolism and new-onset HF) and the progression of cardiac involvement at 3 months, with 14 ng/L as the best cut-off [87]. In patients treated with ICI, guidelines recommend measuring baseline cardiac troponin to stratify cardiotoxicity risk; moreover, it should be monitored in order to detect ICI-induced myocarditis [39]. Less evidence exists regarding the increase in BNP/NT-proBNP as a marker for cancer therapy-related cardiotoxicity [20]. In fact, a BNP/NT-proBNP increase after chemotherapy may be associated with fluid overload, limiting its diagnostic and prognostic role. Elevated BNP levels have been found at baseline in patients with malignancies and without HF and/or sepsis; it is likely that tumor-related mechanisms and oxidative stress contribute to increasing this biomarker [88].

In patients with multiple myeloma receiving proteasome inhibitors, baseline high levels of BNP (>100 pg/mL) and NT-proBNP (>125 pg/mL) predicted cardiovascular adverse events associated with worse overall outcomes [89]. It is recommended to monitor NP at baseline, before starting proteosome inhibitors, and during treatment, especially in the setting of cardiac amyloidosis [39]. Regarding a biomarker-guided cardioprotective strategy, the recently published multicenter prospective Cardiac Care trial failed to demonstrate a cardioprotective effect of a troponin-measurement-guided strategy in patients receiving anthracycline-based chemotherapy [90]. Certainly, the use of cardiac biomarkers shows advantages compared to imaging in the management of cancer patients and, in particular, they are easier to obtain by oncologists and more reproducible than imaging; they are also less time-consuming for patients. However, many problems still limit their implementation in clinical practice, such as the lack of standardization in trial methodologies evaluating biomarkers, the wide heterogeneity in terms of malignancy types, cancer treatment schedules and the definition of cardiotoxicity across the trials.

## 5. Cancer-Therapy-Related Cardiac Dysfunction: Clinical Utility of Biomarkers and the Role of Genetic Polymorphisms

Anthracyclines are the oldest chemotherapeutic drugs known to cause cardiovascular toxicity. Anthracycline-induced cardiotoxicity is cumulative, dose-dependent, irreversible and can present with symptomatic or asymptomatic CTRCD. The baseline measurement of NP and cTn is recommended before receiving anthracycline chemotherapy, especially in high- and very high-risk patients, and before each cycle (in high- and very high-risk patients) or every other cycle during anthracycline chemotherapy and either 3 or 12 months after the completion of cancer therapy [39].

Concerning HER2-targeted therapies, trastuzumab-induced cardiotoxicity has been widely studied and it is largely reversible and dose-independent, suggesting that genetic factors may play an important role in its occurrence [91,92]. An increase in cTn levels identifies patients at higher risk of trastuzumab-induced CTRCD, and the serial measurement of NP was more sensitive in predicting subsequent declines in LVEF during trastuzumab treatment [39]. Fluoropyrimidines such as 5-fluorouracil and its oral prodrug capecitabine represent the second cause of cardiotoxicity from chemotherapy, following that from anthracyclines, although their cardiotoxicity is generally underestimated and underdiagnosed [93]. Vascular endothelial growth factor (VEGF) inhibitors, including monoclonal antibodies and tyrosine kinase inhibitors (TKIs), may cause relevant cardiovascular effects, leading to an impairment in the balance between vasodilation and vasoconstriction, undermining endothelial cell integrity and interacting with off-target pathways [94]. The measurement of NP may be considered in all patients, at baseline and then every 4 months during the first year in moderate-risk patients, or at baseline and 4 weeks after starting treatment, and then every 3 months during the first year, in high- and very high-risk patients [39].

Multitargeted kinase inhibitors targeting BCR-ABL (including imatinib, bosutinib, dasatinib, nilotinib and ponatinib) are associated with unique toxicity, because of the “off-target” effects of each drug [39].

Radiotherapy (RT) can also negatively affect cardiac function by promoting myocardial fibrosis and microvascular damage. The risk of developing heart disease in subjects irradiated to the chest is greater than in the general population. In a prospective study of a cohort of patients with Hodgkin’s or non-Hodgkin’s lymphoma treated with anthracyclines, it was demonstrated that RT may have an additional unfavorable impact on the myocardial longitudinal systolic deformation [95].

The most common forms of CTRCD are summarized in Table 1. Examples of increases in serum biomarkers correlated to cardiotoxicity are listed in Table 2.

Knowledge of the pathophysiology of chemotherapy-induced cardiotoxicity is essential in order to develop strategies aimed at reducing such toxicity. Furthermore, it is important to highlight that the type of damage and the pathways involved can guide the search for genetic polymorphisms associated with a reduction or an increase in the risk of cardiotoxicity. A genetic polymorphism is a variation in the DNA sequence present in at least 1% of the population. It has been shown that genetic polymorphisms can influence traits and susceptibility to diseases or the effectiveness of drug therapies (i.e., the effectiveness of chemotherapy or the possible onset of adverse events in oncological treatments).

In the most recent literature, there are many examples of the application of genetic polymorphisms to identify genetic susceptibility in the onset of cardiac damage related to oncological therapy. A study that included 176 women with breast cancer without concurrent cardiovascular diseases, who were scheduled for polychemotherapy with anthracyclines, recommended the evaluation of genetic polymorphisms in the p53 protein (rs1042522) and NOS3 (rs1799983), NADPH-oxidase (rs4673), GPX1 (rs1050450), ADRB1 (Arg389Gly, rs1801253) and MMP-3 (rs3025058) genes prior to starting chemotherapy. This study shows that the maximum risk of cardiotoxicity is associated with the presence of the p53 protein gene Arg/Arg genotype and NOS3 gene T/T genotype [103]. A genome-wide association study (GWAS) and pathway analysis of changes in LVEF after exposure to anthracyclines in in 385 subjects revealed that the presence of SNP rs10443221 on chromosome 1 (1p32.1) near PRDM2 conferred protection from cardiotoxicity (4.11-point increase in LVEF for each alternate allele) [104]. Numerous pharmacogenomics studies have identified the synonymous genomic variant rs7853758 and the intronic variant rs885004 in SLC28A3 as statistically associated with a lower incidence of anthracycline-induced cardiotoxicity. A study conducted on six well-phenotyped, doxorubicin-treated pediatric patients showed that patient-derived cardiomyocytes recapitulate the cardioprotective effect of the SLC28A3 locus and that SLC28A3 expression influences the severity of doxorubicin-induced cardiotoxicity (DIC). This study provides two potential therapeutic options to attenuate DIC, repurposing FDA-approved desipramine or SLC28A3-AS1 long noncoding RNA therapy, and proposes a simple clinical test to detect the presence of rs11140490 to predict that a patient will be less likely to experience DIC [105]. A case–control association study of 2258 genetic variants between nine cases of trastuzumab-induced cardiotoxicity and controls from the Japanese general population, registered in the Human Genetic Variation Database, identified a novel variant in the EYS gene associated with trastuzumab-induced cardiotoxicity. This finding provides new insights into personalized trastuzumab therapy for patients with HER2-positive cancer [106]. The VEGF-634 CC polymorphism is associated with higher risks of both bevacizumab-induced hypertension and thromboembolism [107]. It has been reported that polymorphisms of the KDR gene decrease VEGF-A’s binding ability to the KDR protein, leading to an increased risk of coronary artery disease [108]. Further studies in this field are needed to aim at personalizing therapeutic schemes also according to the genetic profile of the patient, targeting the treatment and avoiding adverse events.

## 6. Multiparametric Approach Integrating Biomarkers, Imaging and Clinic: The Example of Multiple Myeloma

Biomarkers and imaging play a key role in the early detection and subsequent monitoring of CTRCD in cancer patients, allowing the early detection of cardiac dysfunction before clinical manifestations [84,109,110,111]. In fact, several studies have shown how natriuretic peptide (NP) determination at baseline or NP changes during follow-up are able to predict future CTRCD [17,112,113].

In particular, in patients with multiple myeloma (MM), who are at a high risk of developing cardiovascular (CV) complications, the measurement of NPs before cancer treatment predicts subsequent CV adverse events with good accuracy. In line with this, in a study of 109 patients with relapsed MM, circulating levels of BNP > 100 pg/mL or NT-proBNP > 125 pg/mL before the initiation of carfilzomib were associated with an odds ratio of 10.8 for subsequent CV adverse events [89].

In addition, a finding of particular interest comes from a study of 555 patients with different types of cancer, in which elevated values at baseline of cardiac biomarkers including NT-proBNP and troponin (cTn) were strongly correlated with all-cause mortality. This observation further supports the hypothesis that the presence of subclinical myocardial damage could be directly related to disease progression [26].

However, in the CARDIOTOX (CARDIOvascular TOXicity induced by cancer-related therapies) registry, which enrolled 855 patients treated with different types of therapy, including radiation therapy (RT), both NT-proBNP and cTn elevations at baseline were not associated with the development of severe CTRCD identified as LVEF < 40% or clinical HF [114].

Therefore, based on these results, the ESC cardio-oncology guidelines recommend NT-pro-BNP and cTn dosages in high- and very-high-risk patients; moreover, their measurement should be considered in low- and moderate-risk patients before treatment with proteasome inhibitors. Due to the emerging role of biomarkers in CRTCD identification, there is great interest in the search for new biomarkers that can further support risk stratification in this patient setting. Candidates include myeloperoxidase, galectin-3, micro-ribonucleic acids and immunoglobulin E. However, at present, there is insufficient evidence to support the use of these biomarkers in clinical practice and further studies are needed [39].

In the absence of biomarkers that are applicable to the entire cancer population, the integration of biomarkers and cardiovascular imaging is the best method to detect chemotherapy cardiotoxicity at an early stage. Indeed, the onset of chemotherapy may be followed by myocardial cell damage with the release of troponins and natriuretic peptides, which occurs about 3 months before the clinical manifestation of cardiomyopathy [34,85]. The latter is followed by a reduction in GLS, which precedes a reduction in LVEF and then the appearance of HF clinical symptoms [39].

Indeed, the determination of GLS is recommended before chemotherapy in patients with previous CV disease or at risk of CTRCD. In particular, GLS is a more sensitive and reproducible measure of LV systolic function than LVEF and may identify subclinical cardiac dysfunction before the detection of abnormal LVEF [115,116,117,118]. Indeed, a 15% reduction from baseline in GLS and/or a reduction in circumferential strain during follow-up is indicative of CRTCD or future left ventricular dysfunction [39]. Thus, both the absolute GLS value and relative reduction may be indicators of subclinical CRTCD [119].

This is even more evident in the case of MM; in fact, in addition to the changes in GLS, the Global Myocardial Work Efficiency (GWE), another echocardiographic parameter, can undergo significant changes in the case of CTRCD. Indeed, in MM patients on bortezomib therapy, reduced GWE values can detect early CTRCD but also cardiac adverse events after six months of bortezomib therapy [120].

In the event of poor-quality echocardiographic images or when a specific condition is diagnosed (e.g., hypertrophic cardiomyopathy), CMR is recommended for better risk assessment.

In symptomatic patients (stable angina, limiting dyspnea) with clinical suspicion of coronary artery disease (CAD), functional imaging testing is recommended, especially before the use of anticancer therapies associated with vascular toxicity (e.g., fluoropyrimidines, VEGFi, breakpoint cluster region–Abelson oncogene locus (BCR-ABL), tyrosine kinase inhibitors (TKIs)). Alternatively, in patients with a low to intermediate pretest probability of CAD, coronary computed tomography angiography (CCTA) is a robust alternative modality with high sensitivity to rule out obstructive CAD [39].

In conclusion, the integrated approach of clinical presentation, biomarkers and imaging methods is the best strategy for the recognition and monitoring of CRTCD, allowing early identification and thus timely therapeutic optimization, reducing CRTCD-related adverse events and improving patients’ quality of life.

## 7. Novel Potential Biomarkers

Reliable biomarkers that could predict cardiotoxicity and/or the early onset of CTRCD are not currently available in the clinical setting. However, troponins and natriuretic peptides have been indicated in the last ESC Guidelines on Cardio-Oncology as risk factors to be evaluated as part of the baseline cardiotoxicity risk assessment because of their usefulness as indicators of cardiomyocyte injury, but their predictive capacity for the onset of cardiotoxicity still lacks reliability. Predictive biomarkers for the early and late onset of CTRCD are urgently needed to mitigate the risks associated with cardiac complications [121]

Novel emerging biomarkers were recently evaluated in a CTRCD setting.

- MicroRNAs (miRNAs) [122]: small endogenous single-stranded non-coding RNAs, which act as modifiers of gene expression post-transcriptionally through the binding to protein-coding messenger RNA [123]. The dysregulation of miRNAs has been associated with various diseases, so they are of great interest as biomarkers, particularly due to their properties of being potentially disease-specific, stable, quantifiable and easily extracted from a range of clinical samples. Several studies show that circulating miRNAs are correlated with CTRCD, especially in breast cancer and in patients treated with anthracyclines and trastuzumab [124]. However, further studies are needed to accurately evaluate the potential use of miRNAs in this clinical setting.
- Myeloperoxidase (MPO): myeloid-lineage-restricted enzyme with bactericidal properties, found in the azurophilic granules of neutrophils, involved in the neutrophil extracellular traps (NETs) that are implicated in myocardial infarction and in serious cardiovascular events [121]. Elevated circulating levels of MPO were found in breast cancer patients that experienced cardiotoxicity, so MPO is now considered a promising biomarker for the early detection of anthracycline-related and anthracycline–trastuzumab cardiac dysfunction, based on the results of several studies [125,126].
- Galectin-3 (Gal-3): a β-galactoside-binding protein and a member of the lectin family, implicated in various pathophysiological processes including fibrosis, inflammation and oxidative stress and known to induce cardiac fibroblast proliferation and collagen production and deposition [127]. Recent studies have investigated Gal-3 as a potential diagnostic biomarker for cancer-therapy-induced cardiac dysfunction in breast cancer patients [37,128,129]. In particular, some studies show changes in the circulating levels of Gal-3 in response to treatment with cardiotoxic breast cancer therapies [37], without clear evidence of an association between elevated levels of Gal-3 and the incidence of cardiotoxicity [129]. Its prognostic impact has not yet been fully understood.
- C-Reactive Protein (CRP): an inflammatory marker assessed as a biomarker for the detection of CTRCD. In the past, various studies have shown a change in CRP levels in response to chemotherapy in breast cancer patients, with no clear association with the onset of CTRCD [37,130,131]. Other studies have found no association between the levels of CRP and subsequent cardiotoxicity [132].
- Growth Differentiation Factor 15 (GDF-15): a hormonal peptide of the transforming growth factor-β superfamily. Increased concentrations of GDF-15 were noted in cardiomyocytes during ischemia–reperfusion injury and myocardial infarction [133], in response to treatment with anthracyclines and trastuzumab in breast cancer patients [132,134]. A study by Tromp et al. [135] showed a strong and significant association between GDF-15 and changes in left ventricular ejection fraction in late breast cancer survivors after correction for potential confounders. Further studies with a larger number of patients are required to evaluate the predictive role of GDF-15 in CTRCD and overcome inconsistencies.
- Other Biomarkers under Investigation: other potentially promising biomarkers are currently under investigation for their utility in the detection and/or prediction of CTRCD. These include endothelin-1, neuregulin-1, plasma bioactive adrenomedullin (ADM), D-dimer, soluble fms-like tyrosine kinase receptor (sFlt)-1, soluble ST2 (sST2), CK-MB, topoisomerase II α gene (TOP2A), cardiac myosin light chain 1 (cMLC-1), vascular endothelial growth factor (VEGF), placental growth factor (PIGF), procollagen-derived type-I C-terminal-propeptide (PICP), epicardial adipose tissue (EAT) volume, circulating bilirubin, hemopexin, glycated hemoglobin (HbA(1)c), advanced oxidation protein products (AOPP), human resistin and vascular adhesion molecule 1 (VCAM-1) [121].

Recently, Varkoly et al. [136] demonstrated for the first time that circulating RNA virus gene sequences (as orthomyxovirus) could be detected in the blood samples of patients with hematological malignancies or breast cancer, and that there is an association between them and myocarditis and lower LVEF in response to chemotherapy.

In summary, traditional biomarkers (such as troponins and natriuretic peptides) are the most frequently used CTRCD biomarkers in clinical trials and in clinical practice, albeit not universally, and their strengths and limitations are well documented. In particular, they could be indicators of cardiomyocyte injury, with a predictive capacity for the onset of cardiotoxicity of poor reliability. Several promising biomarkers are reported in the literature, with the aim to develop a more advanced and accurate risk stratification model for the prediction and early detection of CTRCD in the cancer population. To date, there is a need for larger additional and prospective clinical studies with sufficient statistical power for the validation of novel biomarkers of CTRCD. In any case, a combination of both serum and imaging biomarkers is encouraged for the best and most precise risk stratification of patients at risk of developing cardiotoxicity in response to cancer therapy.

## 8. Non-Conventional Potential Biomarkers: Omics

To date, conventional cardiotoxicity parameters and biomarkers have typically shown significant changes only after evident heart damage. In recent decades, researchers have shifted their focus to unraveling the mechanisms underlying cardiotoxicity to identify potential biomarkers for early cardiac damage. Omics sciences, including genomics, transcriptomics, proteomics and metabolomics, have been extensively employed in this pursuit. The sensitivity of metabolomics, with its ability to capture the complexity of entire metabolic networks, makes it one of the most promising approaches in the quest for early cardiovascular damage biomarkers [137].

Doxorubicin (DOX) is well known for exhibiting cardiotoxicity at cumulative doses, leading to increased intracellular reactive oxygen species in the heart. Palmer et al. conducted a two-phase study on human-induced pluripotent stem-cell-derived cardiomyocytes (hiPSC-CM) to predict cardiotoxicity development. They identified four metabolites (lactic acid, arachidonic acid, thymidine and 2′-deoxycytidine) with essential roles in regulating mitochondrial function, oxidative stress and replication, associated with cardiotoxicity [138].

Similarly, Dionisio et al. explored cardiotoxicity induced by cyclophosphamide, an anticancer prodrug known to cause cardiotoxicity and other severe adverse effects. Their in vitro metabolomic approach revealed a connection between cyclophosphamide’s active metabolite, 4-hydroxycyclophosphamide, and acrolein. Results indicated that these metabolites led to mitochondrial and lysosomal impairment, increased intracellular sugar levels, affected Krebs cycle metabolites and altered amino acid levels [139].

Li et al. identified 39 biomarkers for the detection of doxorubicin, isoproterenol and 5-fluorouracil cardiotoxicity earlier than biochemical and histopathological analyses in a mouse model. They established a predictive model characterized by L-carnitine, 19-hydroxydeoxycorticosterone, lysophosphatidylcholine (LPC) (14:0) and LPC (20:2) [140].

Schnackenberg’s group discovered 18 metabolites significantly altered in the plasma and another 22 metabolites increased in cardiac tissue after a cumulative doxorubicin dose of 6 mg/kg. The detection of myocardial injury and cardiac pathology, however, was not possible until cumulative doses of 18 and 24 mg/kg, respectively. In both serum and tissue specimens, the levels of many amino acids (including arginine and citrulline), biogenic amines, acylcarnitines (carnitine) and tricarboxylic acid cycle (TCA)-related metabolites (e.g., lactate, succinate) were found to be altered [141].

Tan et al. identified DOX-induced cardiotoxicity constituted by 24 metabolites involved in glycolysis, the citrate cycle and the metabolism of some amino acids and lipids [142].

Jensen et al. demonstrated significant decreases in docosahexaenoic acid, arachidonic acid/eicosatetraenoic acid, o-phosphocolamine and 6-hydroxynicotinic acid in the hearts of mice treated with sunitinib [143], along with alterations in taurine/hypotaurine metabolism, after sorafenib treatment [144].

Unger’s group identified, for the first time, a six-metabolite panel constituted by SM (d18:1/16:0), SM (d18:1/18:0), PC (16:0/14:0), PE (16:0/20:4), 1-(1,2-dihexanoylphosphatidyl) inositol-4,5-bisphosphate and Gly-Arg-Gly-Asp-Asn-Pro. All these metabolites were found to be upregulated in the plasma of patients showing cardiotoxicity, using samples from both rat models exposed to radiation and patients receiving radiation therapy for esophageal cancer [145].

In the context of breast cancer patients—the most studied population—Asnani’s group emphasized the importance of intermediary metabolism in anthracyclines and trastuzumab-treated patients. The authors recognized variations in aconitic and citric acid capable of discriminating patients who developed cardiotoxicity from those who did not. Moreover, citric acid levels correlated with the modification in LVEF at three months and with absolute LVEF values at nine months. Patients with cardiotoxicity also exhibited increased purine metabolites inosine, hypoxanthine and uric acid, while pyrimidine metabolites pseudouridine and orotic acid characterized those patients who did not show cardiotoxicity [146].

Preliminary data from Cocco et al. seem to demonstrate, for the first time, that early anthracycline-induced cardiotoxicity (detected by asymptomatic GLS reduction) is correlated with a characteristic metabolic profile: higher concentrations of Krebs cycle intermediates (fumarate and succinate) and fatty acids (such as linoleic acid) in patients with cardiotoxicity and increased levels of cardioprotective metabolites, such as tryptophan, in patients without cardiotoxicity. Interestingly, the damage induced by cardiotoxicity showed the upregulation of metabolites similar to those identified in clinical HF and mice models of cardiotoxicity [147].

Regarding carfilzomib-related cardiotoxicity, the group of Tantawy demonstrated lower plasma levels of pyruvate and higher values of lactate [148].

More recently, Yang et al. identified mitochondrial-regulated glycerolipid metabolism as a key player in metabolic changes due to immunotherapy-related cardiotoxicity [149].

The discussed studies underscore how metabolomics could represent a sensitive and promising tool in cardio-oncology research. Its translational utilization could lead to a more efficient follow-up in all cardiotoxicity-prone patients.

The Central Figure (Figure 1) summarizes the complex bidirectional relationship between cancer and cardiovascular disease and the major role of widely utilized and novel biomarkers.

## 9. Central Figure

**Figure 1:**
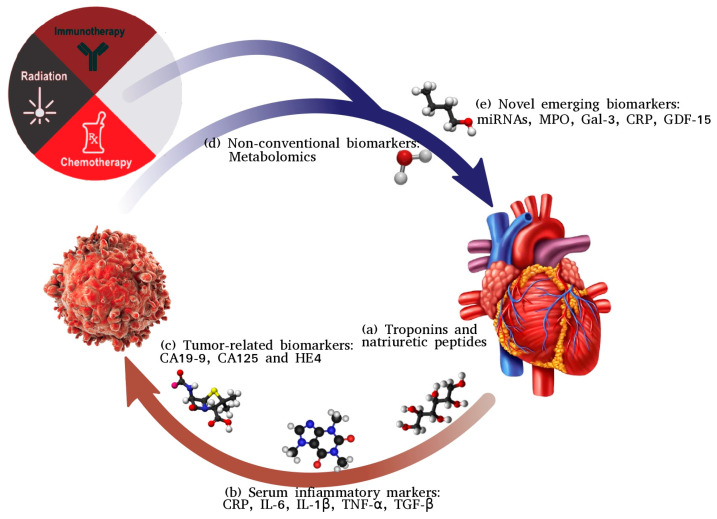
Beyond cancer therapy-related cardiac dysfunction (CTRCD): bidirectional relationship between cancer and heart disease. (**a**) Increased levels of natriuretic peptides and troponins have been found in cancer patients, even before the introduction of anticancer therapy, without evidence of abnormal cardiac function. Malignant cells are capable of producing vasoactive peptides, such as vasopressin or endothelin-1, as well as cardiac neurohormones, such as NPs. (**b**) Cancer and cardiovascular disease share many risk factors, as well as a common underlying inflammatory condition. Some serum inflammatory markers such as C-reactive protein (CRP) and proinflammatory cytokines like interleukin 6 (IL-6) are expressed in both cancer and heart failure. (**c**) Patients affected by HF present with high serum levels in several biomarkers presumed to be tumor-related, such as CA19-9, CA125 and human epididymis protein 4 (HE4). In particular, CA125 has shown prognostic value in different cardiac settings and HE4 is demonstrated to be strongly associated with HF. (**d**) Metabolomics could represent a sensitive and promising tool in cardio-oncology research, including more efficient follow-up in all patients at risk of cardiotoxicity. CTX-induced damage showed upregulation of metabolites similar to those known in clinical heart failure. (**e**) Novel emerging biomarkers in CTRCD setting are needed to mitigate the risks associated with cardiac complications.

## 10. Conclusions

In this literature review, we described the current state of knowledge on the meaning and the usefulness of cardiovascular biomarkers in patients with cancer, taking into account the intricate relationship between cancer and cardiovascular disease and how this affects cardiovascular and tumor biomarkers. Furthermore, we elucidated the role of cardiovascular biomarkers in the risk stratification and in the identification of chemotherapy-induced cardiotoxicity, according to the latest 2022 ESC Guidelines on Cardio-Oncology, providing a summary of the novel potential biomarkers in this clinical setting.

An increase in cardiac biomarkers reflects the hemodynamic overload (natriuretic peptides) and cardiomyocyte damage (high-sensitivity troponins). Cardiac biomarkers are sensitive parameters of cardiac damage. The current ESC cardio-oncology guidelines recommend the evaluation of biomarkers at the baseline evaluation and during follow-up visits, with different recommendations concerning the timing according to the anticancer treatment and baseline risk class. Cardiac damage in cancer patients may be secondary to antineoplastic treatment but can also be the consequence of cardiac damage secondary to pathophysiological mechanisms related to the cancer per se or secondary to cardiovascular comorbidities. Furthermore, cardiac biomarkers can increase in the case of worsening renal function. NT-proBNP is characterized by significant intrinsic variability and only variations greater than 20% should be considered as clinically relevant. A multiparametric approach evaluating cardiac biomarkers (and their changes over time) with other clinical/instrumental parameters (including imaging) can help in the identification of cardiac damage. Cardiac biomarkers allow the ruling out of cardiac involvement in specific onco-hematological settings (i.e., cardiac involvement in multiple myeloma). Novel potential inflammatory and non-inflammatory biomarkers are under investigation in this clinical setting. New omics sciences can identify early signs of cardiac damage in the setting of antineoplastic-drug-induced cardiotoxicity.

## Figures and Tables

**Table 1:** List of the most used chemotherapy classes of agents and their possible cardiovascular toxicity. The boxes are marked if at least one of the compounds, belonging to the respective class, is shown to cause the reported effect with at least a common frequency (≥1/100) according to EMA and FDA prescribing information and to the most recent European guidelines on cardio-oncology [39,96,97].

	Arrhythmia	HF	Vascular Toxicity	VTE/PE	Systemic HTN	Other
Anthracyclines	✓	✓	✓		✓	
Alkylating agents	✓	✓				
Antimetabolites		✓	✓	✓		
Immunomodulatory drugs	✓	✓	✓	✓	✓	DM
Taxanes	✓				✓	
Platinum-based agents			✓	✓		
Androgen deprivation therapy		✓	✓		✓	
Proteasome inhibitors	✓	✓		✓	✓	PH
HER2 inhibitors	✓	✓			✓	
VEGF inhibitors	✓	✓	✓	✓	✓	
BCR-ABL1 inhibitors	✓	✓	✓		✓	Pleuro-pericardial Effusion PH
ALK inhibitors	✓				✓	Dyslipidaemia DM
EGFR inhibitors		✓		✓		
BRAF inhibitors	✓	✓		✓	✓	Bleeding
MEK inhibitors	✓	✓			✓	Bleeding
Immuno-checkpoint inhibitors	✓	✓	✓	✓		Myopericarditis

List of abbreviations: DM, diabetes mellitus; EMA, European Medicines Agency; FDA, Food and Drug Administration; HF, heart failure; HTN, hypertension; PE, pulmonary embolism; PH, pulmonary hypertension; VTE, venous thromboembolism.

**Table 2:** Examples of increased levels of serum biomarkers during specific oncological therapies.

Cancer Treatment	Biomarker of Cardiotoxicity	References
Anthracycline Bevacizumab Cyclosporine A Isoprenaline	- miR-146a, miR-1, miR-133a and miR-208 - troponin I (>80 ng/L) - NT-proBNP (>100 ng/L)	Skála, M. et al. *Arch Toxicol*, 2019 [98]; Cardinale, D. et al. *Circulation*, 2004 [84]; Horie, T. et al. *Cardiovascular Research,* 2010 [99].
Immune checkpoint inhibitors	- TnTc (>1.5 ng/L) - NPs (no clear cut-off)	Mahmood, S.S. et al. *Journal of the American College of Cardiology*, 2018 [100].
CAR-T cell therapies	- TnTc (no clear cut-off)	Lee, D.W. et al. *Blood*, 2014 [101].
Proteasome inhibitors (bortezomib, carfilzomib and ixazomib)	- NPs (no clear cut-off)	Cornell, R.F. et al. *Journal of Clinical* *Oncology*, 2019 [89].
Trastuzumab	- hsCRP (>3 mg) - NPs (no clear cut-off)	Onitilo, A.A., et al. *Breast Cancer Research and Treatment*, 2012 [102].

List of abbreviations: miR-146a, microRNA 146a; miR-1, microRNA-1; miR-133a, microRNA 133a-1; miR-208, microRNA-208; NT-proBNP, N-terminal pro-B-type natriuretic peptide; TnTc, cardiac troponin T; NPs, natriuretic peptides; CAR-T cell, chimeric antigen receptor T-cell; hsCRP, high-sensitivity C-reactive protein.

## Data Availability

Not applicable.
